# Supplementary material for: Can a Forest Tree Species Progeny Trial Serve as an Ex Situ Collection? A Case Study on Alnus glutinosa
Source: Plants (Basel). 2023 Nov 27;12(23):3986. doi: 10.3390/plants12233986 (PMC10708541; doi:10.3390/plants12233986)

The number of alleles found in *A. glutinosa* Vytėnai progeny trial per different allele frequency categories represented for different sample groups

A: allele copy number in one family on average, when single population frequencies are used;
B: allele copy number in six families, when single population frequencies are used;
C: allele copy number in 22 families, when single population frequencies are used;
D: allele copy number in six families, when five populations average frequencies are used;
E: allele copy number in 22 families, when five populations average frequencies are used;
F: allele copy number in 22 families, when 10 populations average frequencies are used.

*families no 103, 121, 135, 139, 89 and 97.

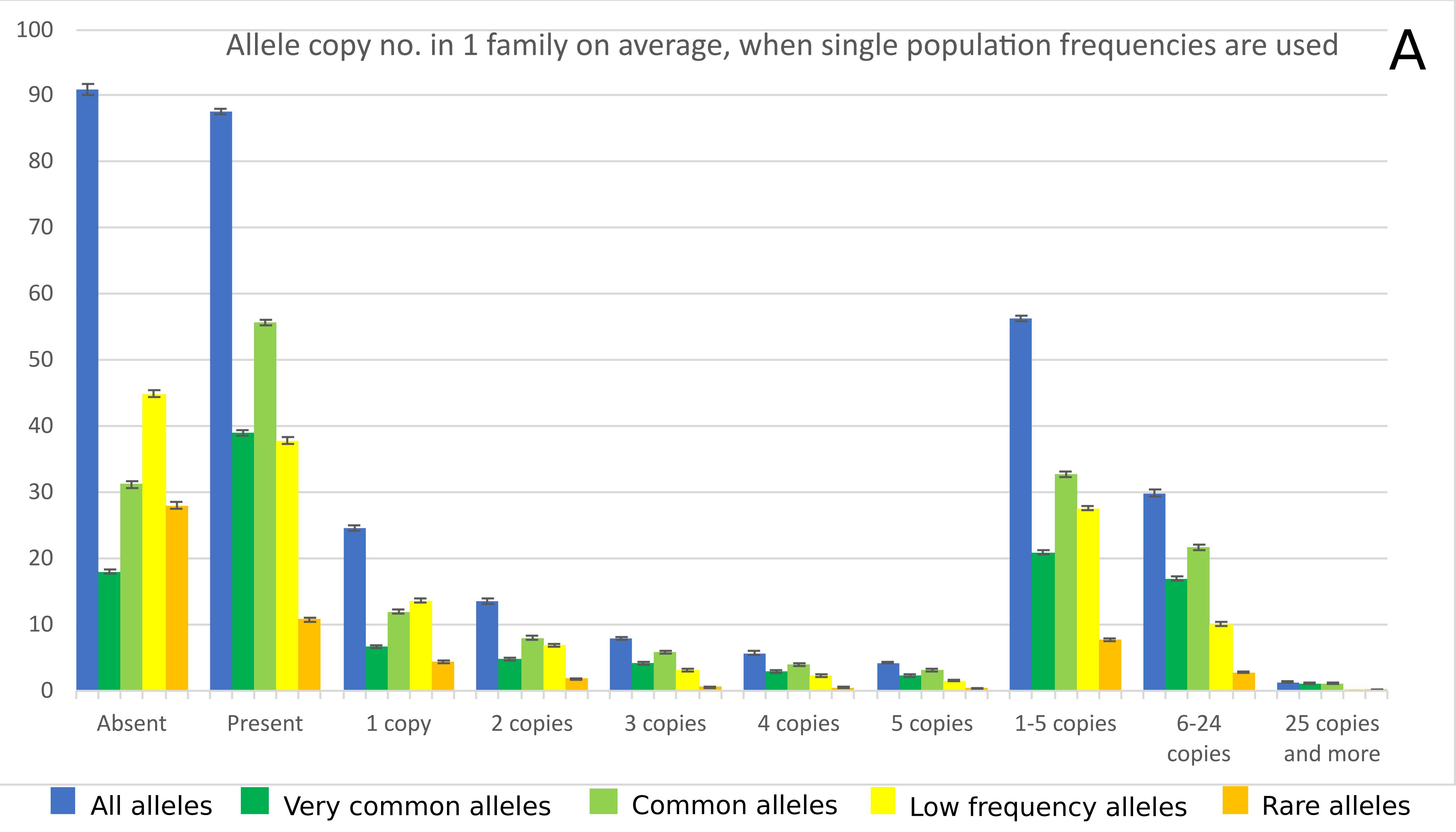

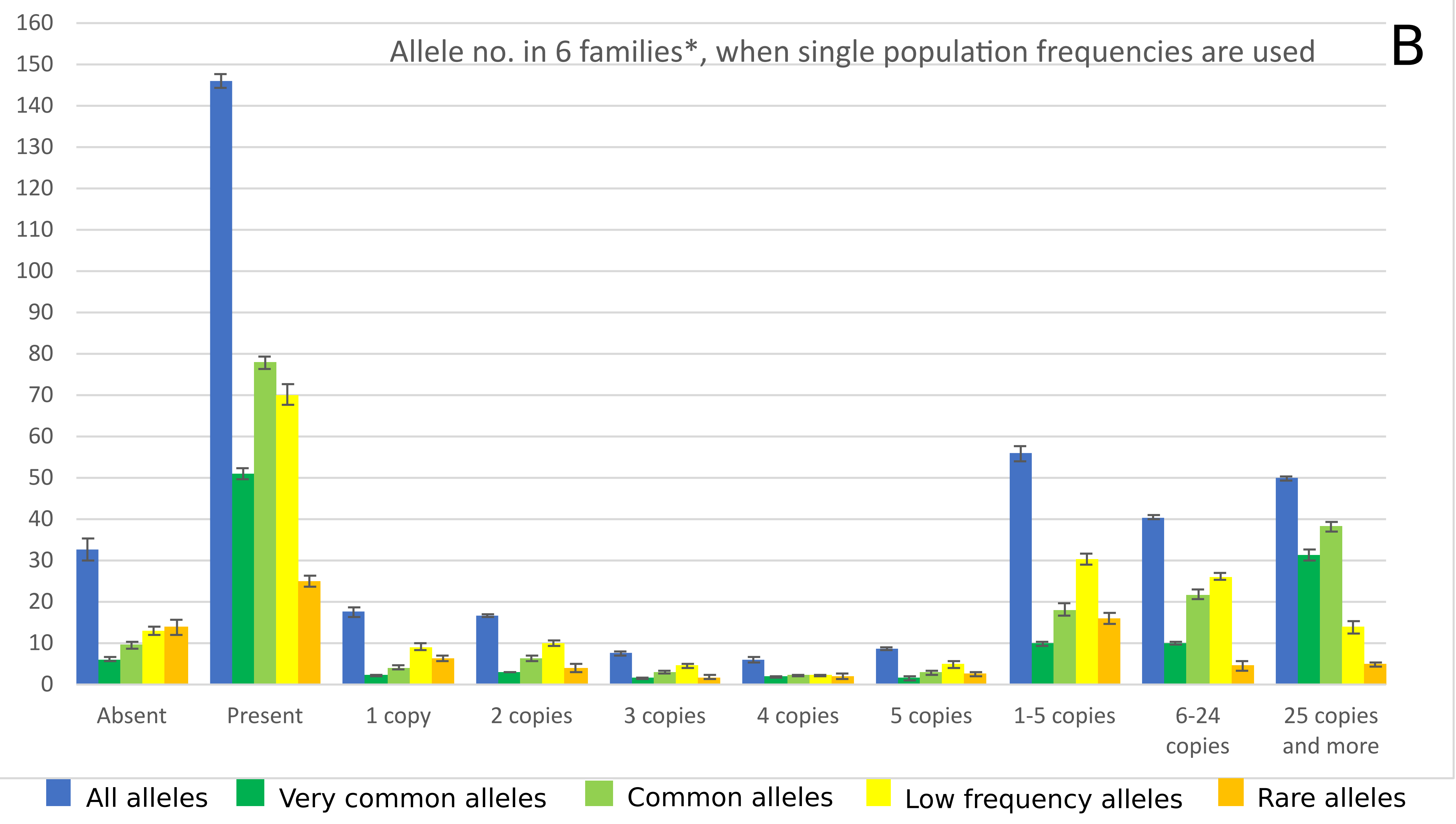

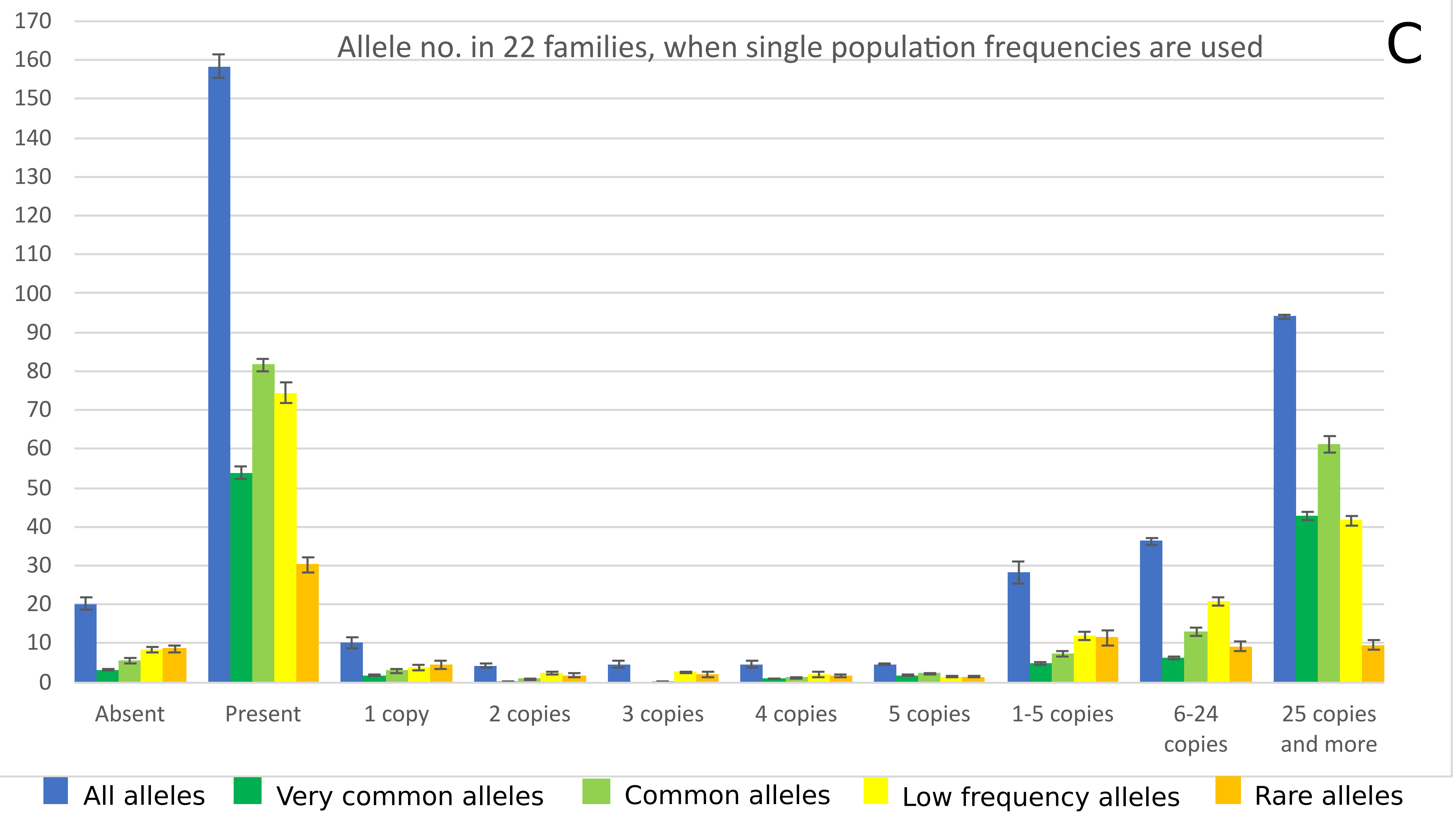

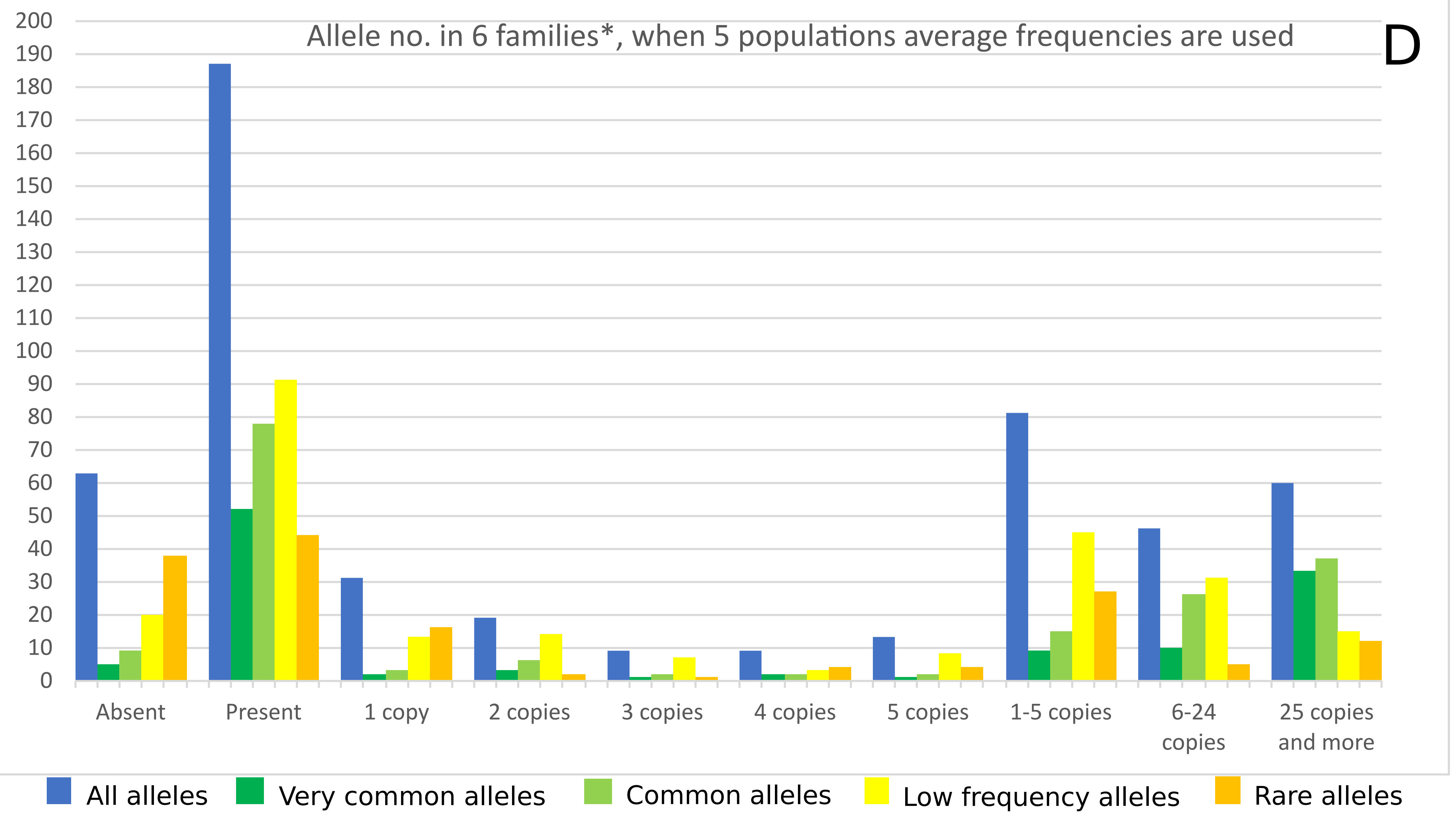

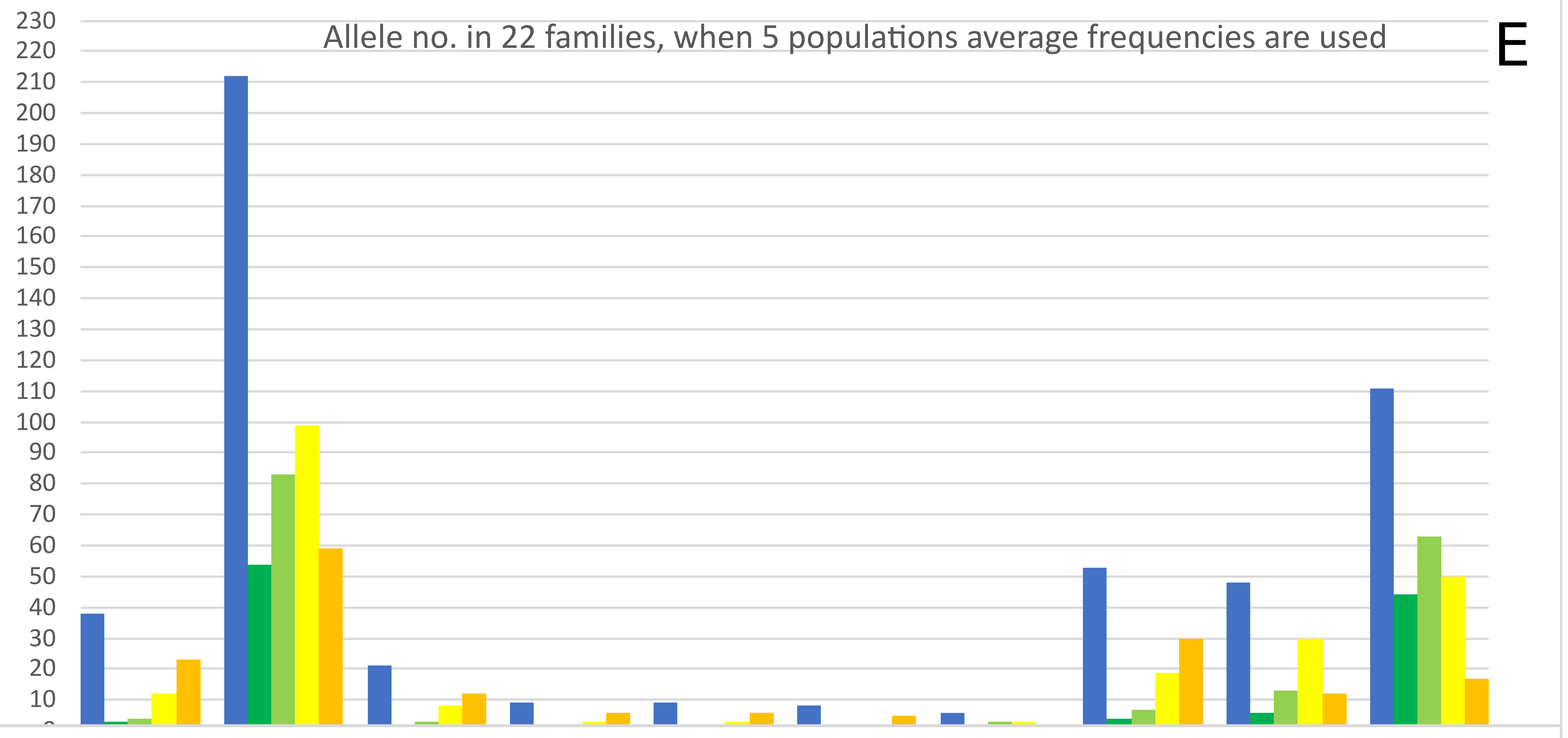

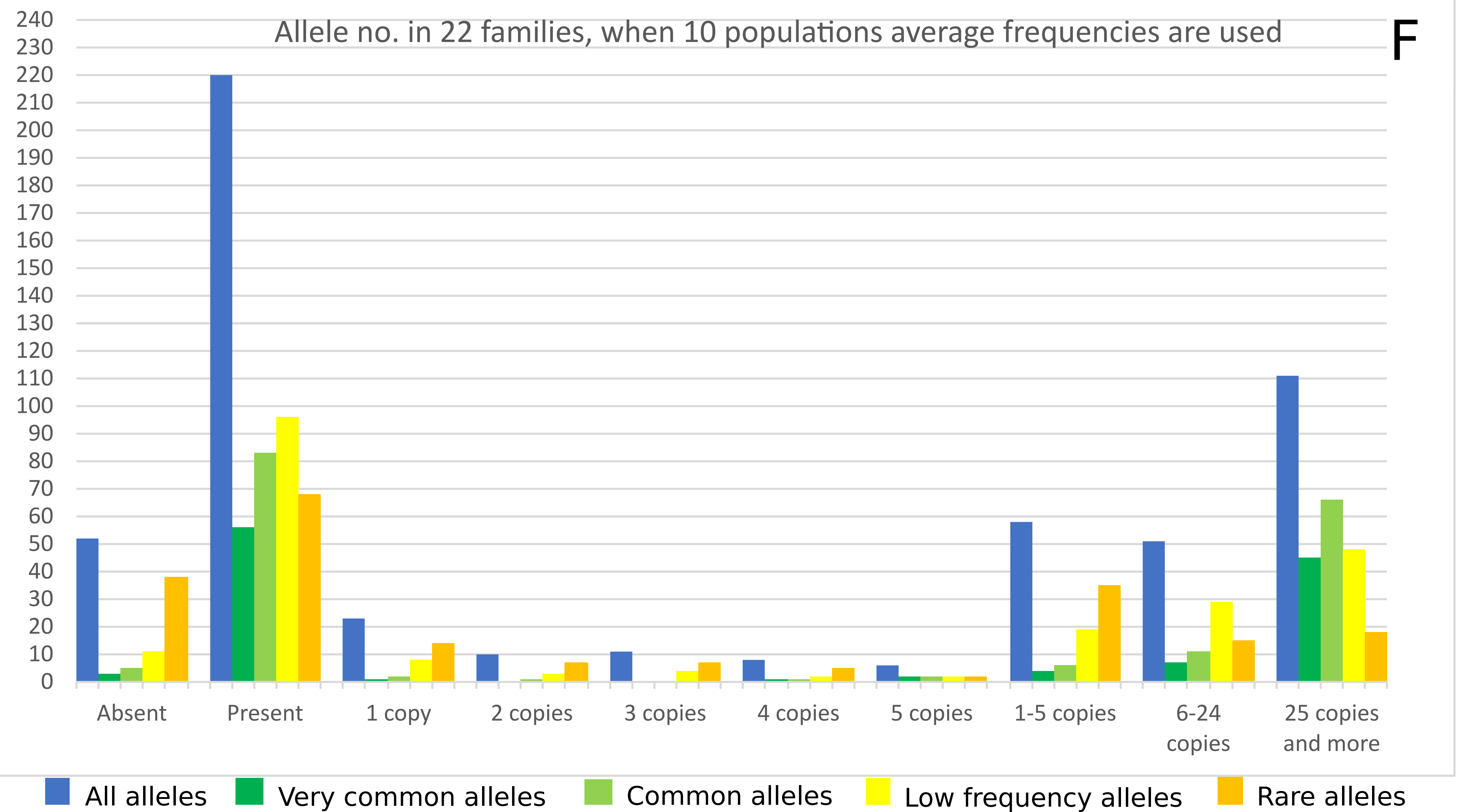

Supplement: Supplementary file 1 [file plants-12-03986-s001.zip › Figure S1.pdf]
