# Supplementary material for: Can a Forest Tree Species Progeny Trial Serve as an Ex Situ Collection? A Case Study on Alnus glutinosa
Source: Plants (Basel). 2023 Nov 27;12(23):3986. doi: 10.3390/plants12233986 (PMC10708541; doi:10.3390/plants12233986)
Supplement: Supplementary file 1 [file plants-12-03986-s001.zip › Table S1.pdf]

Table S1. Genetic diversity parameters of different *A. glutinosa* sample lots (GCU populations and open pollinated families), compared in present study. *Na* – no of different alleles; *Ne* – effective no of alleles; *Ho* – observed heterozygosity; *He* – expected heterozygosity; *F* – inbreeding coefficient; *Ar* – allelic richness, based on 2 diploid individuals. All genetic diversity parameters are provided with standard errors.

Sample lot	*Na*	*Ne*	*Ho*	*He*	F_{IS}	*Ar*
JUO	11.00±0.65	4.98±0.46	0.64±0.04	0.77±0.02	0.18±0.04	2.94±0.08
VIL	11.12±0.65	5.17±0.49	0.76±0.03	0.76±0.03	0.01±0.03	2.92±0.11
LEI	11.24±0.79	5.22±0.60	0.75±0.04	0.75±0.04	0.01±0.04	2.89±0.12
GIR	10.12±0.81	5.01±0.49	0.75±0.03	0.75±0.04	0.00±0.03	2.89±0.11
SIM	10.65±0.61	5.14±0.55	0.74±0.04	0.75±0.04	0.02±0.02	2.90±0.11
PUR	10.24±0.75	4.85±0.54	0.73±0.04	0.73±0.04	-0.00±0.03	2.82±0.13
BAT	10.06±0.64	5.08±0.54	0.73±0.04	0.75±0.04	0.03±0.03	2.89±0.12
PAZ	9.88±0.65	4.60±0.47	0.73±0.04	0.73±0.04	0.01±0.03	2.81±0.11
MIK	10.47±0.71	4.46±0.44	0.70±0.04	0.72±0.04	0.05±0.04	2.79±0.11
SPA	10.35±0.78	5.15±0.55	0.77±0.05	0.74±0.05	-0.03±0.03	2.88±0.13
5 pops combined*	14.71±0.83	5.30±0.51	0.71±0.03	0.77±0.03	0.08±0.02	2.87±0.05
10 pops combined	16.00±0.98	5.37±0.55	0.73±0.03	0.76±0.03	0.04±0.02	2.88±0.03
family 61	6.06±0.53	3.30±0.35	0.67±0.06	0.62±0.05	-0.06±0.05	2.52±0.15
family 64	6.88±0.50	3.67±0.32	0.75±0.06	0.68±0.04	-0.08±0.06	2.66±0.11
family 67	6.35±0.59	3.25±0.31	0.69±0.07	0.62±0.05	-0.05±0.06	2.54±0.14
family 71	6.53±0.46	3.58±0.28	0.85±0.04	0.69±0.03	-0.21±0.05	2.66±0.10
family 78	6.18±0.40	3.37±0.28	0.75±0.05	0.66±0.03	-0.10±0.05	2.59±0.10
family 83	6.71±0.40	3.64±0.28	0.72±0.05	0.68±0.04	-0.02±0.05	2.68±0.10
family 86	6.35±0.51	3.46±0.28	0.81±0.04	0.68±0.03	-0.17±0.05	2.62±0.10
family 89	6.47±0.56	3.38±0.26	0.79±0.06	0.67±0.05	-0.17±0.06	2.59±0.10
family 97	6.41±0.53	3.16±0.30	0.70±0.06	0.63±0.04	-0.10±0.07	2.49±0.11
family 103	6.59±0.56	3.44±0.34	0.72±0.06	0.64±0.05	-0.10±0.05	2.56±0.13
family 104	6.12±0.53	3.26±0.28	0.82±0.05	0.65±0.04	-0.24±0.05	2.53±0.11
family 107	6.18±0.57	3.18±0.38	0.61±0.07	0.59±0.06	-0.01±0.06	2.45±0.16
family 108	7.29±0.48	3.81±0.25	0.83±0.03	0.72±0.02	-0.13±0.05	2.76±0.07
family 112	5.94±0.55	3.69±0.38	0.69±0.08	0.65±0.06	-0.03±0.06	2.64±0.15
family 116	7.00±0.59	3.66±0.28	0.83±0.05	0.69±0.04	-0.18±0.05	2.67±0.11
family 119	6.41±0.42	3.50±0.28	0.81±0.05	0.67±0.04	-0.19±0.04	2.62±0.11
family 121	5.82±0.37	3.31±0.22	0.85±0.05	0.67±0.03	-0.25±0.04	2.59±0.09
family 126	6.53±0.44	3.56±0.26	0.80±0.05	0.68±0.04	-0.15±0.04	2.66±0.10
family 128	5.82±0.54	3.10±0.23	0.79±0.05	0.64±0.03	-0.21±0.06	2.49±0.10
family 132	6.41±0.59	3.71±0.35	0.81±0.06	0.67±0.04	-0.19±0.06	2.63±0.13
family 135	6.82±0.58	3.88±0.26	0.87±0.03	0.72±0.02	-0.19±0.05	2.75±0.08
family 139	6.59±0.51	3.65±0.28	0.82±0.05	0.69±0.04	-0.17±0.05	2.67±0.11
6 families combined**	11.65±0.77	4.88±0.42	0.79±0.04	0.75±0.03	-0.05±0.02	2.61±0.04
22 families cobined	13.65±0.92	5.39±0.47	0.78±0.04	0.77±0.04	-0.01±0.02	2.61±0.02

*JUO, VIL, BAT, PAZ, MIK populations
**families 103, 121, 135, 139, 89, 97
