# Supplementary material for: Can a Forest Tree Species Progeny Trial Serve as an Ex Situ Collection? A Case Study on Alnus glutinosa
Source: Plants (Basel). 2023 Nov 27;12(23):3986. doi: 10.3390/plants12233986 (PMC10708541; doi:10.3390/plants12233986)
Supplement: Supplementary file 1 [file plants-12-03986-s001.zip › Table S2.pdf]

Table S2.. *A. glutinosa* open pollinated families, sampled in the Vytėnai progeny plantation, their origin and the number of samples used in this study. The shaded open pollinated families originate from GCUs investigated in this study.

No.	OP Family/Plus tree no.	No of tree sampled per OP family	SFE regional branch and forest district where the plus tree is located	Coordinates	
1.	103/33JPL103	20	Kazlų Rūda r.b., Šunskai	54°43'5.8764"	22°57'23.961"
2.	104/33JPL104	20	Kazlų Rūda r.b., Varnabūdė	54°35'53.376"	23°37'50.484"
3.	107/34JPL107	16	Šakiai r.b., Gerdžiai	54°57'28.278"	23°20'0.3048"
4.	108/34JPL108	20	Šakiai r.b., Šakiai	55°1'52.914"	23°4'7.4136"
5.	112/14JPL112	18	Panevėžys r.b., Gegužinė	55°48'42.8646"	24°29'31.6674"
6.	116/08JPL116	20	Trakai r.b., Pravieniškės	54°56'26.304"	24°9'19.692"
7.	119/08JPL119	19	Trakai r.b., Pravieniškės	54°52'43.9968"	24°21'48.9312"
8.	121/18JPL121	17	Tauragė r.b., Batakiai	55°20'18.9456"	22°28'16.0962"
9.	126/52JPL126	20	Kazlų Rūda r.b., Kazlų Rūda	54°41'13.56"	23°32'36.4554"
10.	128/29JPL128	20	Druskininkai r.b., Musteikos	53°57'18.3888"	24°24'39.276"
11.	132/51JPL132	20	Dubrava r.b., Vaišvydava	54°49'22.512"	24°7'28.308"
12.	135/35JPL135	20	Kretinga r.b., Juodkrantė	55°36'34.5384"	21°7'55.6998"
13.	139/35JPL139	20	Kretinga r.b., Juodkrantė	55°33'58.32"	21°7'40.44"
14.	61/49JPL61	15	Dubrava r.b., Gaižiūnai	54°58'17.0466"	24°16'12.6012"
15.	64/49JPL64	20	Dubrava r.b., Karmėlava	55°0' 44748"	24°15'5.616"
16.	67/15JPL67	20	Prienai r.b., Dambrava	54°41'54.096"	23°48'49.356"
17.	71/51JPL71	19	Dubrava r.b., Vaišvydava	54°43'4.7316"	23°47'51.1506"
18.	78/51JPL78	18	Dubrava r.b., Ežerėlis	54°51'51.0516"	23°41'27.33"
19.	83/16JPL83	19	Radviliškis r.b., Baisiogala	55°41'20.4102"	23°39'21.4266"
20.	86/20JPL86	19	Kuršėnai r.b., Gruzdžiai	56°1'34.698"	23°5'54.1896"
21.	89/53JPL89	20	Kuršėnai r.b., Pažiužmėliai	56°3'53.5746"	22°44'17.667"
22.	97/36JPL97	20	Telšiai r.b., Kuliai	55°49'32.79"	21°38'33.9246"
