# Supplementary material for: Can a Forest Tree Species Progeny Trial Serve as an Ex Situ Collection? A Case Study on Alnus glutinosa
Source: Plants (Basel). 2023 Nov 27;12(23):3986. doi: 10.3390/plants12233986 (PMC10708541; doi:10.3390/plants12233986)
Supplement: Supplementary file 1 [file plants-12-03986-s001.zip › Table S3.pdf]

Table S3. *A. glutinosa* populations used for genetic diversity comparisons. The shaded GCUs had progenies in Vytėnai progeny trial.

**Abbrev.**	**GCU code**	**GCU**	**No of trees sampled**	**SFE regional branch and forest district**	**Coordinates**	
JUO	35JSM037	Juodkrantė seed stand	58	Kretinga r.p., Juodkrantė	55°36'30.899″	21°7'54.800"
VIL	33JGD008	Žalioji forest black alder genetic reserve	60	Kazlų Rūda r.p., Šunskai	54°43'7.284"	22°57'25.091"
LEI	03JSM049	Leipalingis seed stand	60	Druskininkai r.p., Leipalingis	54°10'37.704"	23°48'10.007"
GIR	32JSM040	Girios seed stand	60	Šalčininkai r.p., Rūdninkai	54°24'44.171"	5°3'15.552"
SIM	17JGD019	Šimkaičiai forest black alder genetic reserve	60	Raseiniai r.p., Šimkaičiai	55°11'40.416"	22°50'9.672"
PUR	31JSM030	Purviniškės seed stand	60	Nemenčinės r.p., Asveja	55°0'55.224"	25°37'39.108"
BAT	18JGD018	Bijotai forest black alder genetic reserve	60	Tauragė r.p., Batakiai	55°20'9.564"	22°28'23.232"
PAZ	53JGD005 /006	Tryškiai forest black alder genetic reserve	60	Kuršėnai r.p., Pažiužmėliai	56°3'28.943"	22°43'11.891"
MIK	60JSM044	Mikoliškės seed stand	58	Kretinga r.p., Kartena	55°47'57.804"	21°29'43.835"
SPA	01JGD001	Biržai Forest black alder genetic reserve	61	Biržai r.p., Spalviškės	56°15'38.159"	24°58'55.308"
