# Supplementary figures and images for: Can a Forest Tree Species Progeny Trial Serve as an Ex Situ Collection? A Case Study on Alnus glutinosa
Source: Plants (Basel). 2023 Nov 27;12(23):3986. doi: 10.3390/plants12233986 (PMC10708541; doi:10.3390/plants12233986)

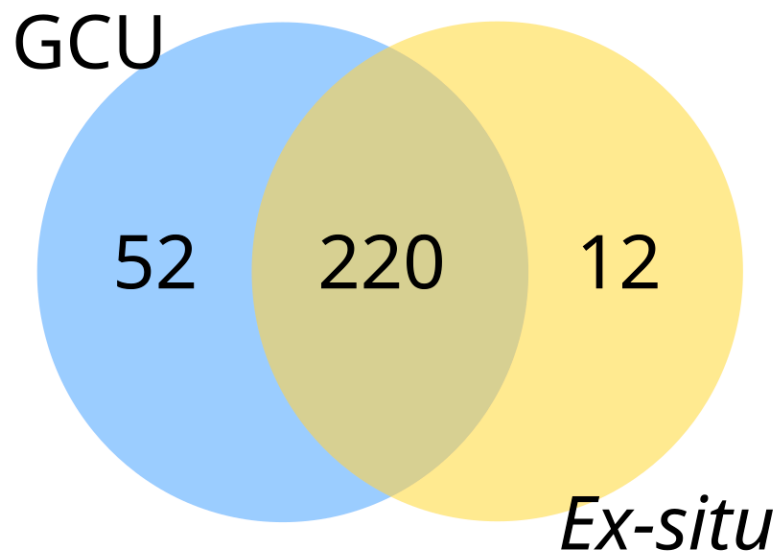

**Figure S2.** Venn diagram of alleles found in natural populations (GCU) and in ex situ plantation.

Supplement: Supplementary file 1 [file plants-12-03986-s001.zip › Figure S2.pdf]
